# Supplementary material for: What choices of visual correction do people make for recreational running?
Source: PLoS One. 2024 Jul 25;19(7):e0305102. doi: 10.1371/journal.pone.0305102 (PMC11271955; doi:10.1371/journal.pone.0305102)
Supplement: Attachment — Submitted filename: Comment for the Editor for PlosONE Manuscript.docx [file pone.0305102.s002.docx]

**Comment for the Editor**

Dear Editor,

Thanks for the opportunity to review this study. It has some novel findings that are relevant to literature. Below are my comments.

**Abstract**

The summary of the method used in the study was not clearly stated in the abstract.

The abstract lacked numbers, percentage values as well as statistical analysis findings that will help for the easy understanding of the study findings.

**Introduction**

The introduction lacked relevant information including justification for the study.

**Method**

The method lacked details.

The authors spent so much time explaining the questionnaire rather than the method.

The study design, population, sample size, and exclusion criteria employed in the study were not clear.

How was the questionnaire developed? Was it validated and how was it validated?

How many participants were recruited and how many ended up participating in the study?

Method used in recruiting participants was not detailed.

Was consent obtained from participant prior to data collection and what type of consent was obtained prior to data collection?

**Results**

The results were confusing and not logically presented.

Most of the information in the tables are redundant and not clear.

I expected the authors to have a table with the characteristics of the study participants.

I struggled to understand the information in table 1 and 2 as well as their purpose and relevance.

The meaning of general purpose in table 2 is not clarified.

The interpretation of the results was simply repetition of what was on the table.

The potential source of bias including confounding variables and how they were adjusted was not clearly stated.

**Discussions**

There was basically no discussion including comparison of the research findings with previous studies.

There was no need to repeat the results in the discussion.

The manuscript had no strengths and limitations.

**Conclusion**

The conclusion does not align with the study objectives.

**Recommendation**

While the paper addresses an interesting issue, it is not publishable in its current form. I therefore recommend rejection.
