# Supplementary material for: What choices of visual correction do people make for recreational running?
Source: PLoS One. 2024 Jul 25;19(7):e0305102. doi: 10.1371/journal.pone.0305102 (PMC11271955; doi:10.1371/journal.pone.0305102)
Supplement: Attachment — Submitted filename: Response to Reviewers.docx [file pone.0305102.s003.docx]

Reviewers' comments:

Reviewer's Responses to Questions

**Comments to the Author**

1. Is the manuscript technically sound, and do the data support the conclusions?

The manuscript must describe a technically sound piece of scientific research with data that supports the conclusions. Experiments must have been conducted rigorously, with appropriate controls, replication, and sample sizes. The conclusions must be drawn appropriately based on the data presented.

Reviewer #1: Yes

Reviewer #2: No

2. Has the statistical analysis been performed appropriately and rigorously?

Reviewer #1: Yes

Reviewer #2: Yes

3. Have the authors made all data underlying the findings in their manuscript fully available?

The [PLOS Data policy](http://www.plosone.org/static/policies.action#sharing) requires authors to make all data underlying the findings described in their manuscript fully available without restriction, with rare exception (please refer to the Data Availability Statement in the manuscript PDF file). The data should be provided as part of the manuscript or its supporting information, or deposited to a public repository. For example, in addition to summary statistics, the data points behind means, medians and variance measures should be available. If there are restrictions on publicly sharing data—e.g. participant privacy or use of data from a third party—those must be specified.

Reviewer #1: Yes

Reviewer #2: No

4. Is the manuscript presented in an intelligible fashion and written in standard English?

PLOS ONE does not copyedit accepted manuscripts, so the language in submitted articles must be clear, correct, and unambiguous. Any typographical or grammatical errors should be corrected at revision, so please note any specific errors here.

Reviewer #1: Yes

Reviewer #2: No

5. Review Comments to the Author

Please use the space provided to explain your answers to the questions above. You may also include additional comments for the author, including concerns about dual publication, research ethics, or publication ethics. (Please upload your review as an attachment if it exceeds 20,000 characters)

Reviewer #1: The is a valuable and interesting study that will be useful in the eye care of runners. The study employed robust statistical methods to achieve its aim of highlighting the choices of visual correction by recreational runners and identifying characteristics associated with them. Though majority use spectacles for general activities, a considerable proportion do not do so when running, unlike those who use contact lenses with nearly equal proportions for both activities. This suggests that there may be problems with use of spectacles for running which the authors rightly identified as an area for future research. Females and younger runners tended to use contact lenses more.

Thank you for your constructive comments.

Few minor revisions could help enrich and improve clarity of the manuscript as follows:
1. In line with scientific writing standards, Line 25 could be rephrased to avoid starting the sentence with a number

Sentence has been rephrased in the Abstract to read:

A 26-item questionnaire covering demographics, running habits, and visual corrections used was answered by 941 people.

2. the results are well presented and mostly clear. A few areas would require clearer presentation. It is suggested that line 198 to 200 be rephrased to convey the finding that spectacles are not worn for running by a proportion of respondents; as presented, it appears as though an inference is being drawn in the results section (which should ideally be done under discussion instead).

Lines 198-200 have been removed from the Results: these points are made in the Discussion at lines 344-350.

3. The discussion section of the manuscript is adequately laid out with interpretation of and elaboration of implications of the study findings as well as relation with existing studies. This section could be made more concise by minimising repetitions. For example, line 320 to 322 which have been expressed in the introduction and methods section and could be deleted.

This text has been deleted as suggested.

Also lines 324 to 325 appear to be repetition of results that had been stated in the results section.

This text has been simplified to read (now lines 337-339):

A strength of the study is the large number (941) of participants sampled, reflecting a broad spectrum of recreational runners whose running demographics represent an ‘above active’ group compared to recommendations (9).

Furthermore lines 332 to 335 contains the same information as line 366 to 369, these could be merged.

Lines 332-335 have been streamlined to remove reference to the potential limitations of spectacles, leaving this point to be made at line 355. This section has also been streamlined to remove re-iteration of figures quoted earlier, and now reads (at line 378):

Although spectacles were the most common correction type for running, a third of the sample (33.1%) actively avoid running in spectacles despite wearing them for general purposes. This finding highlights that spectacles have limitations for a subset of our sample when running as compared to general purpose use. Future analysis of sections 4 & 5 of this questionnaire will consider reasons why people avoid running whilst wearing spectacles.

The manuscript provides a very useful contribution to knowledge about visual correction preferences of recreational runners with visual impairment that would help advise their care. It is recommended that the manuscript be accepted for publication.

Thank you.

Reviewer #2: Abstract
The summary of the method used in the study was not clearly stated in the abstract.
The abstract lacked numbers, percentage values as well as statistical analysis findings that will help for the easy understanding of the study findings.

Word limits restrict the additional information that can be added to the abstract, but it is now additionally specified that an ‘online questionnaire’ was used within the Methods. Percentage values are provided within the Abstract for the key prevalences of visual corrections worn for running. There are insufficient words available to provide odds ratios or P values for all statistics, but named relationships between visual correction choices and demographic features are now described as ‘significantly associated’ with the precise statistical data provided in the Results section of the manuscript.

Introduction
The introduction lacked relevant information including justification for the study.

More specific justification for the study is now provided at line 92:

Given the variety of refractive visual correction choices available and the potential disadvantages of spectacles for sport, we wanted to understand what correction choices are used by people for the popular participatory sport of recreational running.

Method
The method lacked details.
The authors spent so much time explaining the questionnaire rather than the method.

Please see responses below for further specific details added to the manuscript.

The study design, population, sample size, and exclusion criteria employed in the study were not clear.

Further details are now provided in the Methods section, including:

- Study design as a ‘cross-sectional online questionnaire’ (line 104),
- The population of interest is specified by the inclusion criteria (line 135) of ‘aged 18 years or older, consider themselves to be a recreational runner and to need refractive correction for everyday life’.
- Recruitment and sample size have been expanded upon at line 128:

Convenience sampling was used to recruit volunteer participants from the UK, Australia, Canada, and USA. No specific minimum sample size was sought for this exploratory study, but a sampling approach reflecting similar work in this area (5) was undertaken to maximise participation. A contact list of running clubs was collated from the website ‘Time Outdoors’ (timeoutdoors.com) and a total of 93 running clubs were emailed requesting that they share the survey link with their members, in addition to social media marketing. The survey was available on Jisc (Online surveys) from 1^st^ October 2020 to 31^st^ March 2021, and was closed after 2 weeks of null responses.

- Exclusion criteria are made more specific at line 137:

Participants were asked to declare that they met each of these criteria, and potential participants who did not meet the stated inclusion criteria were excluded from completion of the questionnaire. Participants could withdraw from the study at any point by closing their browser window.

How was the questionnaire developed? Was it validated and how was it validated?

The questionnaire was developed to address the aims of the study and therefore covered questions about the participants (section 1), their visual corrections (section 2) and their running (section 3). Since the instrument was designed to be exploratory, rather than a validated outcome measure for assessing any intervention, formal validation was not undertaken. However, piloting was undertaken to assess the instrument for clarity, which is now specified on line 106:

The questionnaire was piloted with five recreational runners to ensure that wording was clear and concise. Following amendment, the questionnaire consisted of five sections and took approximately 20-30 minutes to complete:

How many participants were recruited and how many ended up participating in the study?

Within the Results (line 170) further information is provided by specifying:

The questionnaire was started by 957 people and was fully answered by 941 self-declared recreational runners (S1 File; also available at <https://doi.org/10.25411/aru.25109270.v1>).

Method used in recruiting participants was not detailed.

Please see response above where the social media marketing undertaken is explained in greater detail (line 128).

Was consent obtained from participant prior to data collection and what type of consent was obtained prior to data collection?

Informed consent was obtained. The ethical statement has been recast to provide further detail at line 123 as:

The study was approved by Anglia Ruskin University Research Ethics Committee and was conducted in accordance with the Declaration of Helsinki. Informed electronic written consent was obtained from all participants. Having been provided with information about the study, participants checked a box on the introductory page of the online survey to indicate their consent to take part prior to progressing to complete the survey.

Further information on withdrawal is now also provided on line 139:

Participants could withdraw from the study at any point by closing their browser window.

Results
The results were confusing and not logically presented.

We hope that the changes outlined below have clarified the concerns raised. We do note that Reviewer 1 found ‘the results are well presented and mostly clear’.

Most of the information in the tables are redundant and not clear.

Tables 1 and 2 summarise the key findings of the study, in terms of its aims to characterise the visual corrections used for running and how these differ from those worn for general purposes in everyday life. While key points from the Tables are highlighted in the Results text, the information in these Tables is not redundant.

To address the points regarding clarity, the wording has been amended in Table 1 and the text describing this Table.

Table 1 legend now reads:

**Table 1**. **Cross-tabulation of visual correction(s) worn by participants for general purposes in everyday life compared to when participating in running.**

Table 1 footnotes now include:

None = no visual correction worn.

Headers have been amended in Tables 1 and 2 to read: ‘**Visual correction type(s) worn’, and ‘General purpose wear’.**

Explanatory text at line 189 has been expanded to read:

Table 1 shows the types of visual correction worn by participants for general tasks in their day to day lives (general purpose wear) compared to the visual correction(s) worn when participating in running. Participants could select options of spectacles, contact lenses (CLs) and going without correction (none), along with combinations of these options.

I expected the authors to have a table with the characteristics of the study participants.

The characteristics of the study population are provided in the text at lines 171-180, and it was felt that providing this information in a Table would be redundant.

I struggled to understand the information in table 1 and 2 as well as their purpose and relevance.

Please see the changes to the Tables outlined above, which we hope has clarified the information provided.

The meaning of general purpose in table 2 is not clarified.

Explanatory text at line 183 has been added to read:

Table 1 shows the types of visual correction worn by participants for general tasks in their day to day lives (general purpose wear) compared to the visual correction(s) worn when participating in running.

Changes to the headers in Table 2 have also been clarified with regard to ‘general purpose wear’ as outlined above.

The interpretation of the results was simply repetition of what was on the table.

The Results are interpreted in the Discussion, in keeping with usual presentation and the recommendations of Reviewer 1 that inferences should not be drawn in the Results section. However, to aid the reader in interpreting the relatively complex Tables, key points from the Tables are drawn out and presented within the text of the Results.

The potential source of bias including confounding variables and how they were adjusted was not clearly stated.

No adjustments were made for confounding variables. Multicollinearity was considered in the logistic regressions, although no values were problematic. This is now specified at line 166:

Any tolerance values <0.1 or variance inflation factors (VIF) >10 would be flagged for further investigation with regard to multicollinearity (17).

Potential sources of bias are now considered in the Discussion at line 337:

A strength of the study is the large number (941) of participants sampled, representing a broad spectrum of recreational runners whose running demographics represent an ‘above active’ group compared to recommendations (9). A limitation of the study is that the volunteers choosing to take part in the study were self-selected, and thus may not fully represent all recreational runners who need to wear visual correction.

Discussions
There was basically no discussion including comparison of the research findings with previous studies.

We agree with the Reviewer in that there is very little previous work in this area to which these results can be compared, which we have added as a strength of the study at line 337 (see below). The findings are compared to the work of Zeri (reference 5) at lines 383-393.

There was no need to repeat the results in the discussion.

Changes to the Discussion have been made to reduce repetition from Results as outlined in response to Reviewer 1.

The manuscript had no strengths and limitations.

Consideration of this point is now added in the Discussion at line 337:

A strength of the study is the large number (941) of participants sampled, reflecting a broad spectrum of recreational runners whose running demographics represent an ‘above active’ group compared to recommendations (9). A further strength of the study is its novelty, with very few studies existing with which the present results can be compared. A limitation of the study is that the volunteers choosing to take part in the study were self-selected, and thus may not fully represent all recreational runners who need to wear visual correction.

Conclusion
The conclusion does not align with the study objectives.

The study objectives are defined at line 93 as:

… we wanted to understand what correction choices are used by people for the popular participatory sport of recreational running. The aim of the current study was to identify the choices of correction (spectacles, contact lenses, no correction, or a combination of these options) made by those who participate in recreational running and require a distance vision correction, and to examine whether there are distinguishing demographics which are associated with particular choices of correction.

The conclusions have been amended at line 416 to read:

We have investigated the choices of visual refractive correction made by those who participate in recreational running and require a distance vision correction. Key findings are that recreational runners are prepared to run in any form of visual correction. Spectacles are the most common solution for running, but a substantive portion choose to avoid wearing them to run. People are motivated out of spectacles if they have access to contact lenses, but if they do not have contact lenses and have a low refractive error, many run without correction.

We hope that these revisions prove satisfactory, and look forward to hearing from you in due course.

Yours sincerely,

Keziah Latham (Prof)
