# Supplementary material for: What choices of visual correction do people make for recreational running?
Source: PLoS One. 2024 Jul 25;19(7):e0305102. doi: 10.1371/journal.pone.0305102 (PMC11271955; doi:10.1371/journal.pone.0305102)
Supplement: Attachment — Submitted filename: Comments to the Editor of PLOSONE.docx [file pone.0305102.s004.docx]

I have no more comments.
